# Broader application of robotic platform to complex mitral cases

**DOI:** 10.1016/j.xjtc.2023.08.024

**Published:** 2023-09-15

**Authors:** Yuji Kawano, Thomas MacGillivray

**Affiliations:** Department of Cardiac Surgery, MedStar Washington Hospital Center, Washington, DC

**Figure:**
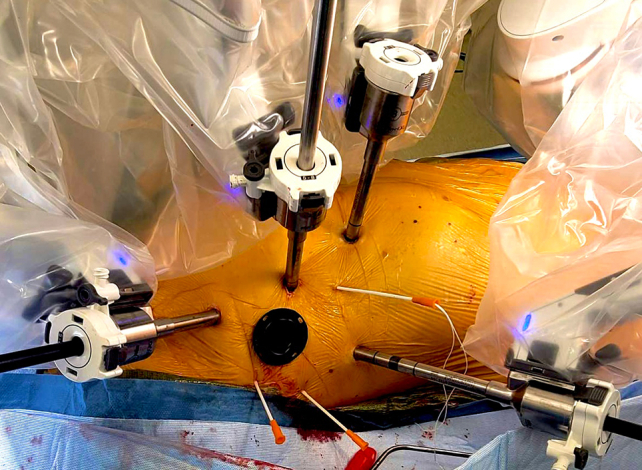
Our standard port-only setting for robotic mitral surgery.

**Central Message**

A robotic platform can be applied safely and efficiently to a more complex patient population. Broader application should be encouraged proactively but carefully to maximize its benefit.

The robotic-assisted approach in cardiac surgery has expanded during the past decades, and it is attracting more and more attention. Nevertheless, robotic mitral surgery constitutes only a small percentage of total cases performed nationally,^1^ and its application is generally limited to relatively simple cases. Patients with challenging features, such as morbid obesity, pectus excavatum, or previous operations, are usually considered unfavorable for robotic surgery. It cannot be emphasized enough that the key for a successful robotic cardiac surgery program is appropriate patient selection. In fact, excellent outcomes have been reported by enforcing strict selection criteria for robotic cardiac surgery.^2^^,^^3^ However, great outcomes have also been reported in more challenging populations by pursuing a liberal approach to include more complex patients.^4^^,^^5^ Since these outcomes were reported from the largest robotic cardiac surgery programs in the nation, it is safe to say that the best patient selection criteria to make the most benefit of robotic approach are yet to be determined. We present several patients with complex conditions who successfully underwent robotic-assisted operations at our institution. All these patients were discharged home without any complications (institutional review board no.: STUDY00006943; September 11, 2023; need for individual consent waived). We believe that robotic instrumentation can be applied to complex patient population safely, when performed by an experienced surgical team. In conclusion, robotic-assisted approach was extremely effective for each one of the presented cases, despite their complicated characteristics. The benefit of this approach could be potentially maximized by encouraging its broader application to more complex patient populations.

## Conflict of Interest Statement

The authors reported no conflicts of interest.

The *Journal* policy requires editors and reviewers to disclose conflicts of interest and to decline handling or reviewing manuscripts for which they may have a conflict of interest. The editors and reviewers of this article have no conflicts of interest.

## Supplementary Data

Video 1 We present several patients with complex conditions who successfully underwent robotic-assisted operations at our institution. Video available at: https://www.jtcvs.org/article/S2666-2507(23)00304-8/fulltext.
