# Supplementary figures and images for: Broader application of robotic platform to complex mitral cases
Source: JTCVS Tech. 2023 Sep 15;22:103–4. doi: 10.1016/j.xjtc.2023.08.024 (PMC10750817; doi:10.1016/j.xjtc.2023.08.024)

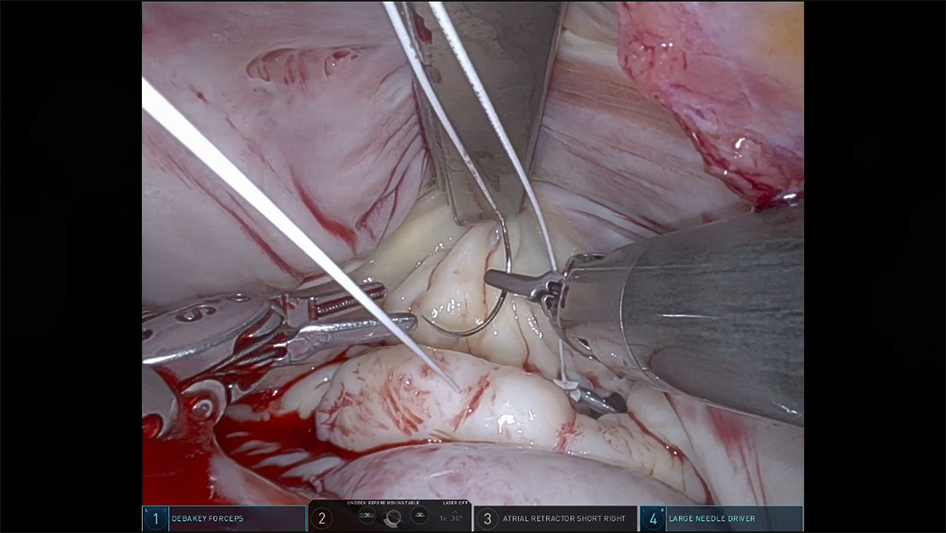

Supplement: Video 1 — We present several patients with complex conditions who successfully underwent robotic-assisted operations at our institution. Video available at: https://www.jtcvs.org/article/S2666-2507(23)00304-8/fulltext. [file fx2.jpg]
